# Sex-based differences in myocardial gene expression in recently deceased organ donors with no prior cardiovascular disease

**DOI:** 10.1371/journal.pone.0183874

**Published:** 2017-08-29

**Authors:** Kolsoum InanlooRahatloo, Grace Liang, Davis Vo, Antje Ebert, Ivy Nguyen, Patricia K. Nguyen

**Affiliations:** 1 Stanford Cardiovascular Institute, Stanford University School of Medicine, Stanford, California, United States of America; 2 Department of Medicine, Division of Cardiology, Stanford University School of Medicine, Stanford, California, United States of America; 3 Department of Genetics, Genetic Research Center, University of Social Welfare and Rehabilitation Sciences, Tehran, Iran; 4 Institute of Stem Cell Biology and Regenerative Medicine, Stanford University School of Medicine, Stanford, California, United States of America; 5 Department of Medicine, Division of Cardiovascular Medicine, Stanford University and Veterans Affairs Palo Alto, Palo Alto, California, United States of America; Mayo Clinic, UNITED STATES

## Abstract

Sex differences in the development of the normal heart and the prevalence of cardiomyopathies have been reported. The molecular basis of these differences remains unclear. Sex differences in the human heart might be related to patterns of gene expression. Recent studies have shown that sex specific differences in gene expression in tissues including the brain, kidney, skeletal muscle, and liver. Similar data is limited for the heart. Herein we address this issue by analyzing donor and post-mortem adult human heart samples originating from 46 control individuals to study whole-genome gene expression in the human left ventricle. Using data from the genotype tissue expression (GTEx) project, we compared the transcriptome expression profiles of male and female hearts. We found that genes located on sex chromosomes were the most abundant ones among the sexually dimorphic genes. The majority of differentially expressed autosomal genes were those involved in the regulation of inflammation, which has been found to be an important contributor to left ventricular remodeling. Specifically, genes on autosomal chromosomes encoding chemokines with inflammatory functions (e.g. CCL4, CX3CL1, TNFAIP3) and a gene that regulates adhesion of immune cells to the endothelium (e.g., VCAM1) were identified with sex-specific expression levels. This study underlines the relevance of sex as an important modifier of cardiac gene expression. These results have important implications in the understanding of the differences in the physiology of the male and female heart transcriptome and how they may lead to different sex specific difference in human cardiac health and its control.

## Introduction

Sex differences in cardiovascular health and disease have been described in multiple clinical observational studies [1]. Previous studies have shown that males and females may vary in the development of the cardiovascular system, resulting in differences in heart size, contractility, and calcium handling [2–4]. Several studies have also demonstrated that sex may play a key role in the susceptibility to various cardiovascular conditions. For example, pulmonary hypertension and heart failure with preserved ejection fraction display a female bias [5, 6], whereas, dilated cardiomyopathy and myocarditis have a male predilection [7, 8]. While many of these clinical differences have been attributed to the presence or absence of sex-specific hormones, namely, estrogen and testosterone [9], a molecular basis for these differences has not been well defined.

Because gene expression is an important determining factor of cellular phenotype, genome-wide transcriptome analysis has been a mainstay of genomics and biomedical research, providing insights into the molecular events underlying human biology and disease. Comparison of the transcription profile between sexes in different tissues such as skin, brain, lung, muscle, and blood has shown widespread differences between males and females. Surprisingly, few studies have described the sex-based differences in both myocardial gene expression in patients without cardiovascular disease. To address this limitation, we analyzed RNA-Seq data obtained from the left ventricle collected from “non-diseased” recently deceased donors in the genotype tissue expression study (GTEX study) [10].

## Material and methods

### RNA-Seq data set

To study the effect of impact of sex on gene expression in the cardiovascular system, we used the RNA-Seq data set produced by the GTEx consortium (dbGaP accession number: phs000424.v1.p1) that aimed to study human gene expression and regulation in multiple tissues [11]. The entire dataset contains RNA-Seq samples obtained from 544 individuals of both European (≈ 84.3%) and African (≈13.7%) descent and includes samples from various tissues including 190 samples from the left ventricle. We used a subset of data from 46 individuals (29 men and 17 women) who had samples from the left ventricle, who were of European descent, who had no prior documentation of cardiovascular disease, who did not take cardiovascular related medications, and whose cause of death was not related to cardiovascular disease (e.g., stroke, myocardial infarction).

Details on library preparation and sequencing, as well as the data QC pipeline, have been previously described [11]. Briefly, using standard non-strand specific protocol with poly-A selection of mRNA (e.g., the Illumina Tru Seq™ protocol which is a large automated protocol implemented at the Broad Institute), RNA samples meeting QC criteria were sequenced on Illumina HiSeq 2000 instruments. Sequence coverage included a minimum of 50M reads (corresponding to a minimum of 25M 76bp paired-end reads).

### Processing, alignment, and analysis of GTEX sequencing data

A copy of the RNA-Seq raw files was downloaded from NCBI dbGaP repository using Sequence Read Archive Tool Kit (SRA Toolkit v2.5.1). Extracted reads were subsequently aligned to the USC human reference genome assembly hg19 and the human transcriptome sequences (Ensembl v70) using the ultrafast aligner the Spliced Transcripts Alignment to a Reference software (STAR, v. 2.3.0e) with maximum tolerated mismatches set at 10 and the outSAMstrandField intronMotif option enabled [12]. Transcriptome coordinates from mapped reads were converted into genomic coordinates and compared with genome mapped reads to identify reads that map to a unique genome location. This process ensured mapped reads were unique across both the genome and transcriptome, while permitting reads to map to different transcripts of the same gene in the initial transcriptome mapping. Uniquely mapped sequences were inspected using the Integrated Genome Viewer (IGV 1.3.1, Broad Institute) [13]. The number of reads uniquely mapped to each gene were counted using Python-HTSeq count module (version 0.5.3p9) [14]. Differential expression analysis was performed on the counts data using DESeq2 (version 1.1.0, http://www.bioconductor.org/packages/release/bioc/html/DESeq2.html). The DESeq2 package uses a negative binomial model to test for differential expression and a shrinkage estimator for the distribution's variance as detailed previously [15]. Briefly, after read counts were retrieved for each gene, differential gene expression was compared using the DEseq2 package in the following four groups: 1) males and females in the entire cohort, 2) younger males and females <55 years, 3) older males and females ≥ 55 years, and 4) younger (<55 years) and older (≥55 years) within male and female cohorts. Read counts for the male and female were compared to determine the log_2_-fold change in abundance of each transcript. Raw p-values were then adjusted for multiple testing with the Benjamini-Hochberg procedure. Genes with adjusted p-value of 0.05 or less were termed as differentially expressed genes. The analysis was performed using R 2.15 and R 3.1.3.

### Enrichment analysis

To determine molecular networks and canonical signaling pathways that contained the differentially expressed genes, we performed a pathway and network enrichment analysis using the Ingenuity Pathway Analysis (IPA) tool (www.ingenuity.com) by inputting a list of differentially expressed genes between male and female heart tissues. Genes with a 1.5 fold or greater change in expression between the male and female groups were used. The settings for the core analysis were as follows: Ingenuity Knowledge Base; Endogenous Chemicals not included; Direct and Indirect relationships. We determined sex-biased enrichment of several genes annotated by KEGG, Reactome, and BioCarta pathway databases with relevance to cardiovascular health and disease. Fisher's exact test was performed for analysis of canonical signaling pathways. The p-value calculated for a pathway showed the probability of being randomly chosen from all of the curated pathways and was adjusted for multiple hypothesis testing using the Benjamini–Hochberg method. IPA was also used to predict which transcription factors could be responsible for gene expression (upstream regulators option) and whether those transcription factors are activated or inhibited. A corrected p*-*value of less than 0.05 was used to define significance.

## Results

### Patient characteristics

The clinical information of the 46 subjects is included in Table 1 and S1 Table. There were no significant differences in the demographic and clinical characteristics between males and females in the entire cohort and in the subset analysis stratified by age (p-value <0.05).

**Table 1:** Demographic and clinical variables of cohort.

		All Groups		Young (<55 years old)		Old (≥ 55 years old)	
Demographic		Male (n = 29)	Female (n = 17)	Male (n = 25)	Female (n = 10)	Male (n = 4)	Female (n = 7)
	Age (years)	42.7±13.3	50.5±14.0	40.0±11.9	42.5±12.8	60.7±4.3	61.9±4.7
	Caucasian (%)	96.7	82.4	96	80	100	85.7
	BMI (kg/m2)	26.8±4.1	26.2±3.9	26.±4.4	28.1±4.2	27.8±2.2	23.6±1.1
**Clinical factors (%)**							
	Hypertension	27 (8/29)	47 (8/17)	20(5/25)	50(5/10)	75(3/4)	43(3/7)
	Heart disease	0	0	0	0	0	0
	COPD/Asthma	20(6/29)	6(1/17)	24(6/25)	10(1/10)	0	0
	Autoimmune, inflammatory, or infectious disease	12(3/29)	0	8(2/25)	0	25(1/4)	0
	Kidney disease	0	6(1/17)	0	0	0	10(1/7)
	Steroid use	0	0	0	0	0	0
**Cause of Death (%)**							
	Hemorrhage	23(7/29)	29(5/17)	24(6/25)	50(5/10)	25(1/4)	0
	Blunt injury	6(2/29)	12(2/17)	4(1/25)	10(1/10)	25(1/4)	14(1/7)
	SIGSW	6(2/29)	0	8(2/25)	0	0	0
	EtOH abuse	3(1/29)	0	4(1/25)	0	0	0
	Hepatorenal syndrome	3(1/29)	0	4(1/25)	0	0	0
	Shock secondary to burns	3(1/29)	0	4(1/25)	0	0	0
	Trauma	6(2/29)	6(1/17)	8(2/25)	10(1/10)	0	0
	Smoke inhalation-respiratory disease	3(1/29)	0	4(1/25)	0	0	0
	Hanging	12(3/29)	6(1/17)	8(2/25)	10(1/10)	25(1/4)	0
	Drug intoxication	12(3/29)	0	8(2/25)	0	25(1/4)	0
	Trauma	6(2/29)	6(1/17)	8(2/25)	10(1/10)	0	0
	Asphyxiation	6(2/29)	6(1/17)	8(2/25)	0	0	14(1/7)
	Seizure	3(1/29)	0	4(1/25)	0	0	0
	Drug overdose	0	18(3/17)	0	10(1/10)	0	29(2/7)
	Complications from ALS	0	6(1/17)	0	0	0	14(1/7)
	Liver transplant	0	6(1/17)	0	0	0	14(1/7)
	Not available	0	6(1/17)	0	0	0	14(1/7)

ALS, amyotrophic lateral sclerosis; MVA, motor vehicle accident; SAH, subarachnoid hemorrhage; SIGSW, survivors of self-inflicted gunshot wound.

### Differential gene expression based on sex and age

**Fig 1:**
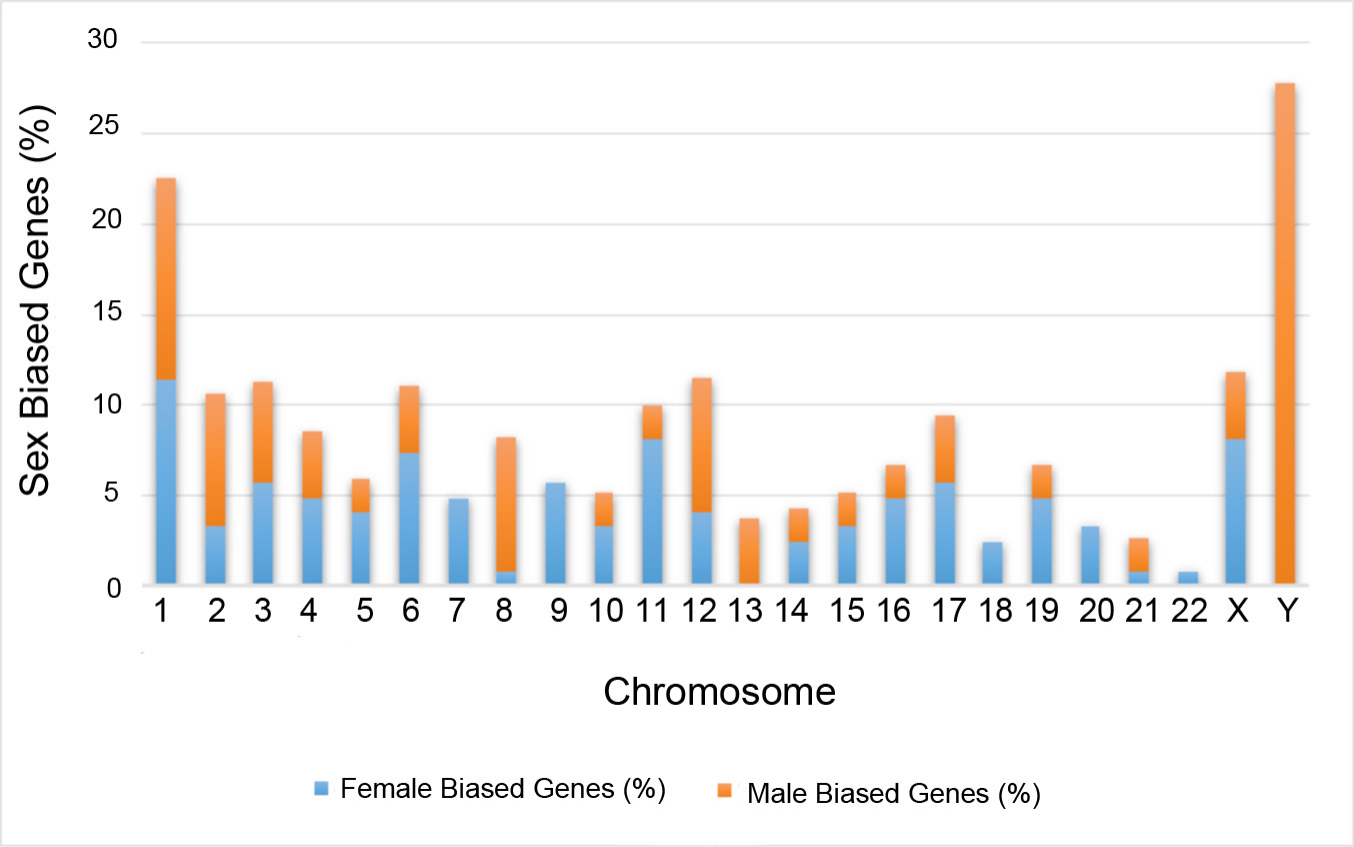
Chromosomal enrichment. For each chromosome, the number of male- and female-biased genes was computed, the table below the graph lists chromosomes that passed the test for enrichment by Fisher exact test with Benjamini correction, P < 0.05.

**Fig 2:**
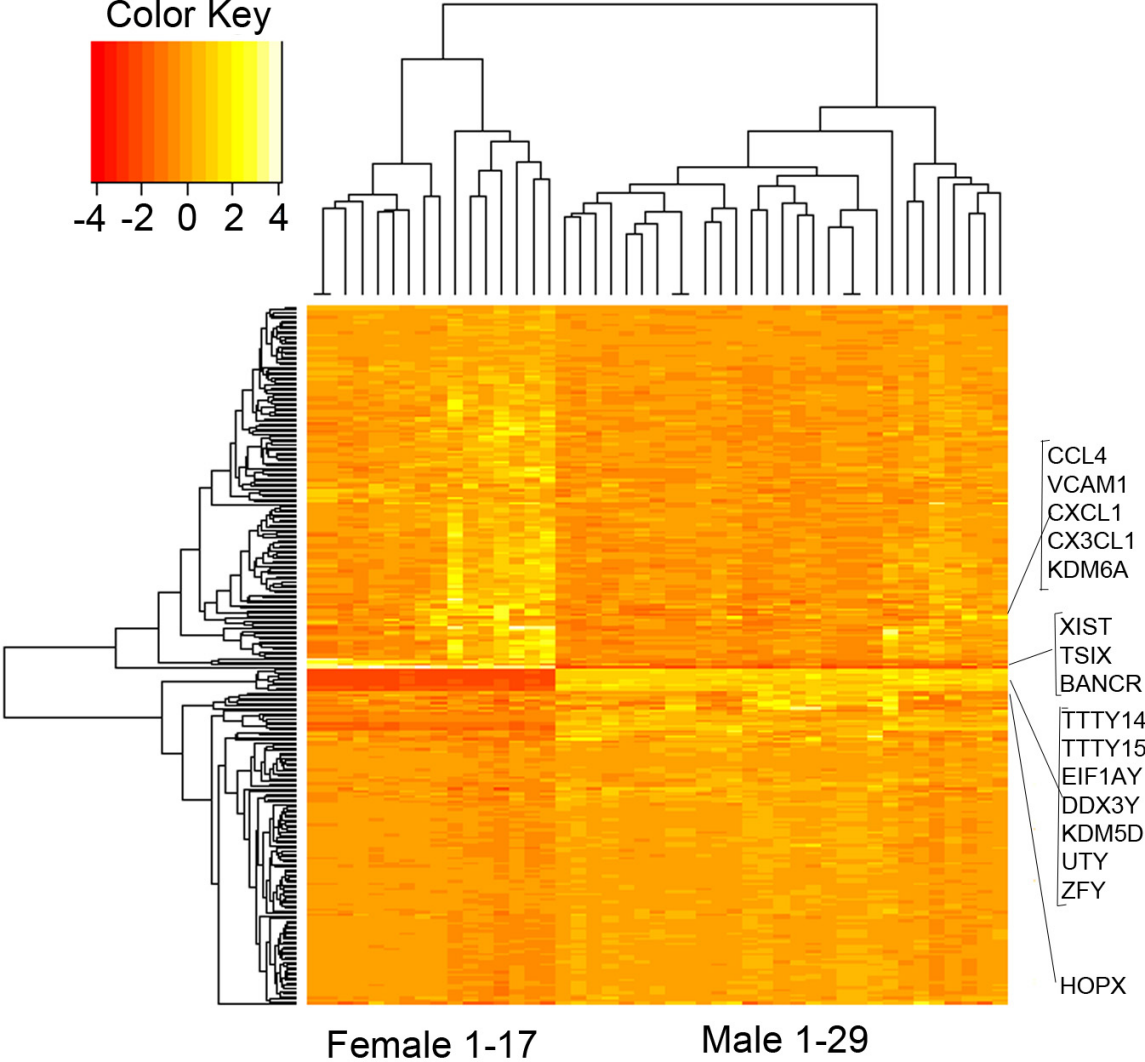
Differentially expressed genes heat map. Heat map showing differentially expressed genes between male and female LV tissues.

Using a fold change of greater than 1.5 and a cut-off of adjusted p-value (q-value) of 0.05 as defined by the Benjamini–Hochberg procedure, we observed that 178 genes were differentially expressed between the left ventricular tissue obtained from male and female donors. Among genes that were differentially expressed, 124 genes were up regulated in females and 54 genes were up regulated in males (S2 Table, Figs 1 and 2), suggesting a significantly larger number of female- compared to male-biased genes. Similar to previous studies in other tissues obtained from “normal” individuals, the differences in gene expression were relatively modest (e.g., the absolute fold difference for most genes was between 1.5 and 2.0) except for X- and Y-linked genes (Table 2).

**Table 2:** Age and sex stratified comparisons of differentially expressed genes, q value <0.05 and fold change > 1.5.

Sex-Dependent Differences	Absolute Fold Change	Differentially Expressed Genes	Female-Biased Genes	Male-Biased Genes
**All Samples (n = 46)**	**1.5–2.0**	138	105	33
	**2.0–2.5**	22	17	5
	**>2.5**	18	2	16
**< 55 (n = 32)**	**1.5–2.0**	5	4	1
	**2.0–2.5**	9	7	2
	**>2.5**	20	4	16
**≥55 (n = 14)**	**1.5–2.0**	9	3	6
	**2.0–2.5**	6	3	3
	**>2.5**	22	4	18

Fig 1 shows the distribution of differentially expressed genes in all chromosomes in women and men. The majority of sex-biased genes were autosomal but, as expected, many were located on the sex chromosomes. *Xist* (X inactive specific transcript) was the sex chromosome linked gene with the highest expression. In addition, eight of these sex chromosome linked genes identified in a previous small microarray study (n = 6) were also differentially expressed in the left ventricular tissue in our cohort (e.g., EIF1AY, RPS4Y1, DDX3Y, JARID1D, USP9Y, ZFY, PRKY, UTY) [16]. Of the autosomal genes not located on a sex chromosome, *HOPX*, a gene that modulates cardiac development, had the highest expression levels. We then compared the frequencies of significant genes on each chromosome against the total number of genes interrogated on each chromosome. Chromosome 11 was found to have more female-biased differential expression, while chromosomes 8 and Y contained more male-biased genes (Fig 1). To determine the effects of age on gene expression levels, we performed an analysis between the older (≥ 55) and younger cohort (<55). Zero genes met the cutoff for differential expression (q value <0.05 and absolute fold change > 1.5) in the female cohort compared with 2 autosomal genes in the male cohort that were up-regulated at least 2.5 fold in younger compared to older men (S3 Table**)**. Based on an analysis of gene ontology pathways, one of these two genes was involved in cytokine activity while the other one encodes for an olfactory receptor that interacts with odorant molecules in the nose.

### Enrichment analysis

To better understand the biological function of genes that were differentially expressed between men and women, sex-biased genes with a q-value < 0.05 and a fold change higher than 1.5 were submitted to IPA for functional annotation and identification of up-stream regulators and canonical pathways. The significant gene ontology (GO) terms associated with each group (male- or female-biased genes) are listed in Tables 3 and 4.

**Table 3:** Biological functions associated with female biased genes.

Network	Top Functions	p Value	Genes
Diseases and disorders			
1	Inflammatory response	4.70E-03–1.08E-08	50
2	Inflammatory disease	4.67E-03–1.39E-07	31
3	Cancer	4.66E-03–2.35E-07	110
4	Gastrointestinal disease	4.67E-03–2.35E-07	101
5	Organismal injury and abnormalities	4.67E-03–2.35E-07	111
Molecular and cellular functions			
1	Cellular movement	4.70E-03–3.85E-11	44
2	Cell-to-cell signaling and interaction	4.40E-03–1.93E-08	35
3	Cell death and survival	4.70E-03–4.11E-07	50
4	Cell morphology	3.34E-03–2.03E-06	28
5	Cellular growth and proliferation	4.69E-03–1.48E-05	49
Physiological system development and function			
1	Hematological system development and function	4.70E-03–3.85E-11	40
2	Immune cell trafficking	4.70E-03–3.85E-11	33
3	Cell-mediated immune response	4.70E-03–2.85E-08	19
4	Tissue morphology	4.70E-03–1.63E-07	43
5	Cardiovascular system development and function	3.34E-03–2.10E-07	29

**Table 4:** Biological functions associated with male biased genes.

Network	Top Functions	p Value	Genes
Disease and disorders			
1	Respiratory disease	1.33E-02–1.47E-04	6
2	Connective tissue disorders	4.58E-02–2.03E-03	5
3	Skeletal and muscular disorders	4.10E-02–2.03E-03	6
4	Cancer	4.58E-02–2.03E-03	5
5	Muscular disorders	4.10E-02–2.03E-03	6
Molecular and cellular functions			
1	Cell morphology	4.80E-02–2.16E-04	13
2	Cell cycle	4.58E-02–3.74E-04	3
3	Cell death and survival	4.58E-02–7.28E-04	5
4	Cell-to-cell signaling and interaction	4.58E-02–2.23E-03	8
5	Cellular assembly and organization	4.58E-02–2.23E-03	7
Physiological system development and function			
1	Connective tissue development and function	4.58E-02–2.16E-04	5
2	Organismal development	4.58E-02–2.16E-04	10
3	Tissue morphology	4.80E-02–2.16E-04	14
4	Auditory and vestibular system development and function	4.37E-02–2.23E-03	2
5	Embryonic development	4.37E-02–2.23E-03	9

This analysis revealed a female bias in the expression of 30 genes related to the immune system (Table 5). In contrast, male biased genes were related to respiratory disease and connective tissue disorders, but did not reach statistical significance (data not shown).

**Table 5:** Female up-regulated genes in inflammatory and heart development pathways.

Term	P-value	Adjusted P-value	Z-score	Combined Score	Genes
Regulation of leukocyte activation (GO:0002694)	5.27207E-06	0.004336	-2.5289	13.75908	SPN; LAG3; CD83; VCAM1; CCL21; FOXF1; FES; CCL5; ZC3H12A; TNFAIP3; THY1; TNFRSF4
Regulation of cytokine production (GO:0001817)	4.10711E-05	0.011260	-2.4979	11.20706	SPN; NFKBIA; AFAP1L2; LAG3; CD83; ZC3H12A; CASP1; TNFAIP3; TNFRSF4; CX3CL1; MB21D1; MAPK13
Taxis (GO:0042330)	3.90027E-05	0.011260	-2.3959	10.74952	SPN; VCAM1; CCL21; CCL5; CCL4; CXCL1; SLIT3; TREM1; CX3CL1
Chemotaxis (GO:0006935)	3.90027E-05	0.011260	-2.3933	10.73764	SPN; VCAM1; CCL21; CCL5; CCL4; CXCL1; SLIT3; TREM1; CX3CL1
Cell chemotaxis (GO:0060326)	5.62173E-05	0.013211	-2.2419	9.699928	VCAM1; CCL21; CCL5; CCL4; CXCL1; TREM1; CX3CL1
Negative regulation of immune system process (GO:0002683)	0.00013572	0.020580	-2.4380	9.467821	SPN; NFKBIA; LAG3; CCL21; FOXF1; ZC3H12A; TNFAIP3; THY1; NBL1
Regulation of inflammatory response (GO:0050727)	0.000155026	0.021251	-2.4038	9.257948	SPN; FOXF1; CCL5; CASP1; TNFAIP3; CX3CL1; CFB; MAPK13
Inflammatory response (GO:0006954)	0.000111624	0.020402	-2.3622	9.193863	AOC3; AFAP1L2; VCAM1; CCL21; CCL5; CCL4; TNFAIP3; CXCL1; TNFRSF4; PTGES
Regulation of immune effector process (GO:0002697)	0.000240717	0.030460	-2.4394	8.51707	SPN; LAG3; FOXF1; FES; TNFAIP3; TNFRSF4; CFB; MB21D1
Regulation of lymphocyte activation (GO:0051249)	0.000282146	0.032930	-2.4716	8.436548	SPN; LAG3; CD83; VCAM1; CCL21; CCL5; TNFAIP3; THY1; TNFRSF4

**Fig 3:**
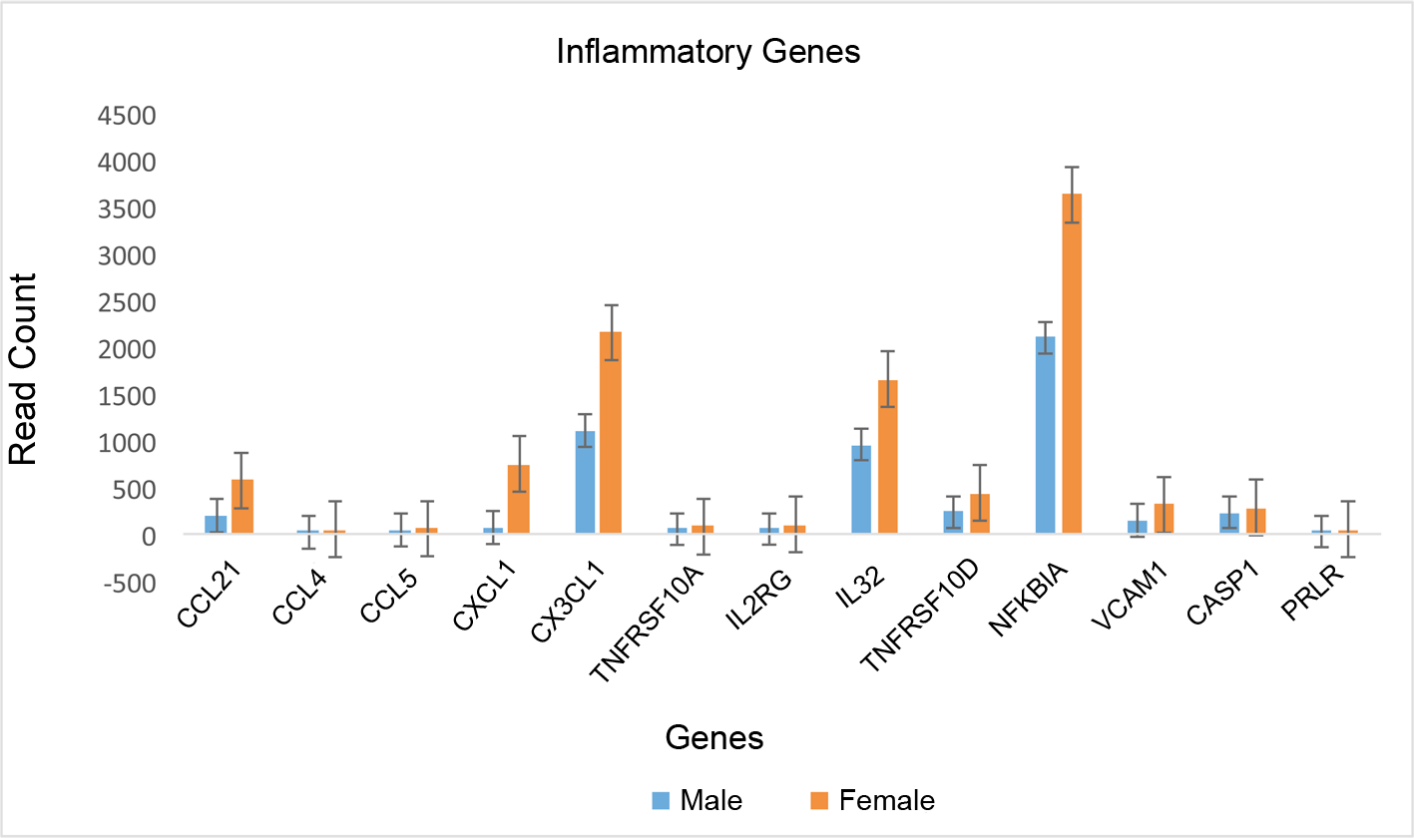
Female heart over expressed genes involve in inflammatory pathways. The bar graphs show the read count and error bars represent standard deviation.

Similarly, analysis of canonical pathways showed significant female bias in the up-regulation of immune-related processes (Fig 3) including the role of IL-17A in arthritis, granulocyte adhesion and diapedesis, and cytokine production; whereas, the top canonical pathways regulated by male-biased genes were hepatic fibrosis and hepatic stellate cell activation (Table 6).

**Table 6:** Five top canonical pathways for female and male biased genes.

**Female-biased top canonical pathways**	**p-value**	**Overlap**
Granulocyte adhesion and diapedesis	5.17E-05	4.0% (7/177)
Agranulocyte adhesion and diapedesis	7.81E-05	3.7% (7/189)
Differential regulation of cytokine production in macrophages and T helper cells by IL-17A and IL-17F	1.20E-04	16.7% (3/18)
Role of IL-17A in arthritis	2.11E-04	7.4% (4/54)
Differential regulation of cytokine production in intestinal epithelial cells by IL-17A and IL- 17F	2.55E-04	13.0% (3/23)
**Male-biased top canonical pathways**	**p-value**	**Overlap**
Hepatic fibrosis / hepatic stellate cell activation	7.31E-04	2.2% (4/183)
Pancreatic adenocarcinoma signaling	1.71E-03	2.8% (3/106)
Glutathione biosynthesis	6.68E-03	33.3% (1/3)
ILK Signaling	8.12E-03	1.6% (3/185)
LPS/IL-1 mediated inhibition of RXR function	1.30E-02	1.4% (3/220)

To verify these findings, we used another public domain enrichment analysis tool, Enrichr (http://amp.pharm.mssm.edu/Enrichr/) [17], to analyze the function of sex-biased genes. GO analysis using Enrichr revealed that genes up-regulated in females heart were involved mainly in biological processes related to immune function including regulation of leukocyte activation, cytokine production and cell adhesion; whereas, no genes were significantly up-regulated in males heart (Table 7).

**Table 7:** Top biological functions, molecular functions and cellular components of female biased genes.

Female-Biased Genes	P-value	Adjusted p-value	Z-score
**Biological function**			
Regulation of cell adhesion (GO:0030155)	1.185E-06	0.0019494	-2.4626837
Regulation of leukocyte activation (GO:0002694)	5.2721E-06	0.00433628	-2.528899
Regulation of cell activation (GO:0050865)	1.0921E-05	0.00598837	-2.5255131
Regulation of cytokine production (GO:0001817)	4.1071E-05	0.01126033	-2.4979698
Mesodermal cell differentiation (GO:0048333)	0.00013762	0.02058018	-2.7762975
**Molecular function**			
Chemokine activity (GO:0008009)	2.2994E-05	0.00738119	-2.4379607
CCR chemokine receptor binding (GO:0048020)	0.00029438	0.0314983	-3.1141924
Chemokine receptor binding (GO:0042379)	5.4359E-05	0.0087247	-2.2605308
**Cellular component**			
Side of membrane (GO:0098552)	1.8363E-05	0.00131292	-2.2865759
External side of plasma membrane (GO:0009897)	2.9874E-05	0.00142397	-2.2381773
Extracellular space (GO:0005615)	1.3675E-05	0.00131292	-2.2091901

Similarly, KEGG metabolic pathway analysis indicated that genes were only up-regulated in female hearts. These genes were located specifically in the pathways of cytokine cytokine receptor interaction (p value = 0.00005, adjusted p value = 0.0023, Z score = -2.08). Similarly, MGI Mammalian Phenotype indicated that mutations of genes in female over-expressed genes were associated with disease in immune system; whereas, there were no significant male over-expressed genes (Table 8). Taken together, genes that were overexpressed in female heart tissue were highly enriched in genes involved in inflammation.

**Table 8:** 5 top MGI mammalian phenotype for female biased genes.

Female-Biased Genes	P-value	Adjusted P-value	Z-score
Abnormal innate immunity	0.0002497	0.016636772	-1.6699686
Abnormal adaptive immunity	8.7305E-05	0.009906402	-1.3827691
Abnormal immune cell	9.2153E-05	0.009906402	-1.3722959

### Transcription factor binding site analysis

**Fig 4:**
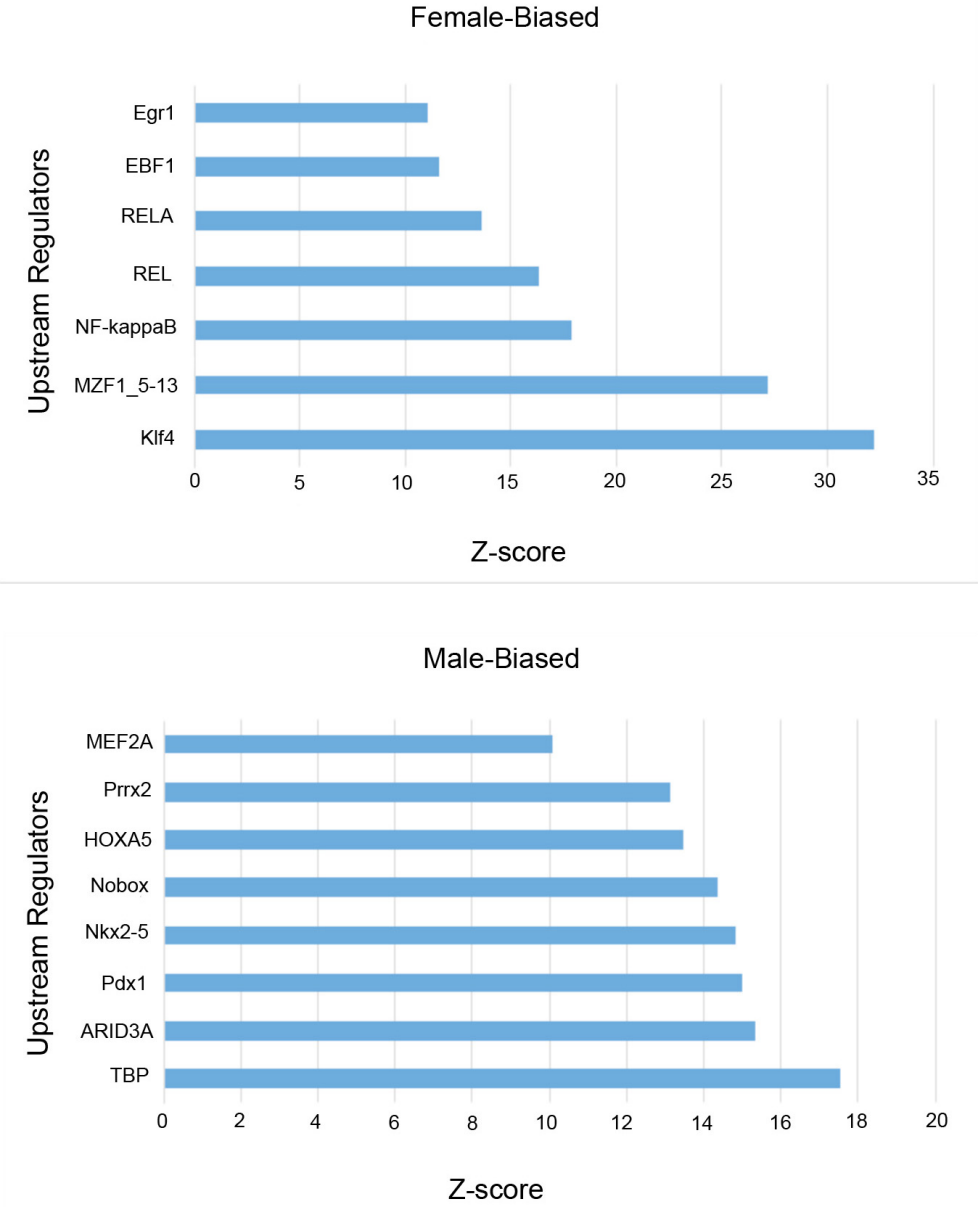
Transcription factor binding sites. Transcription factor binding site analysis comparing male-biased and female-biased genes in human LV tissue.

**Fig 5:**
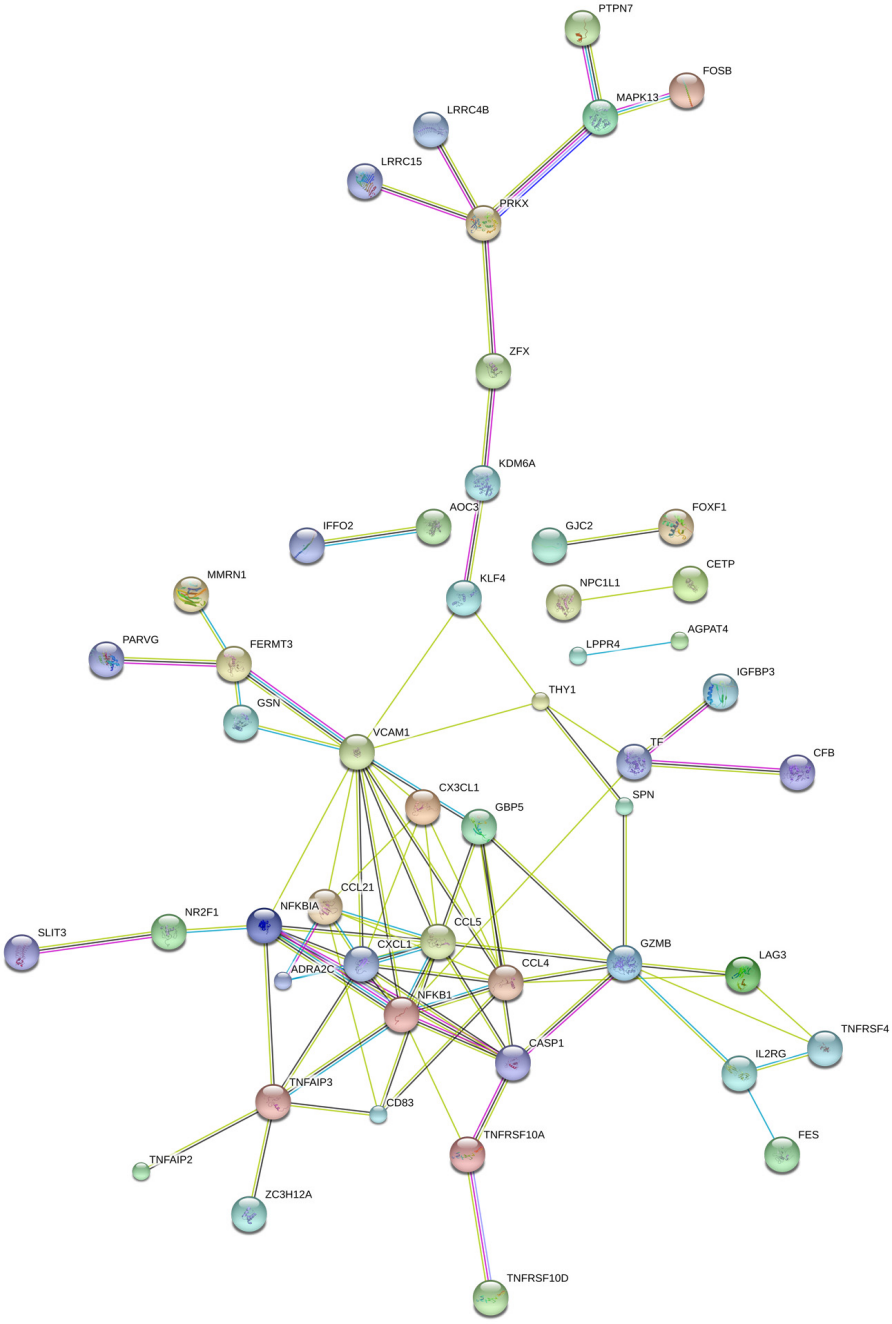
Associations between DEGs and predicted TFs. Diagram of the network of predicted associations between DEGs and predicted TFs (KLF4 and NF-kappa B). The network nodes are proteins. The edges represent the predicted functional associations.

To predict transcription factors that may be involved in controlling sex-biased gene expression, we searched for conserved transcription factor binding sites (TFBS) in the 5 kb of DNA sequence up and downstream of the transcription start sites of sex-biased genes defined previously. Using the web-based promoter analysis program, oPOSSUM-3 and the JASPAR core motifs [18, 19], we identified potential binding sites for 7 transcription factors for female over expressed genes and 8 transcription factor for male over expressed genes (S4 Table, Fig 4) (Z-score > 10; Fisher score> 7). We tested if the genes encoding these TFs were expressed at detectable levels in the human heart tissue using publicly available RNA-Seq data (http://medicalgenomics.org/). Two transcription factors, KLF4 and NF-kappa B, were expressed at detectable levels in human heart tissues. These transcription factors regulate the expression of several genes involved in the regulation of the immune system that were differentially expressed between male and female donor hearts (S4 Table, Fig 5).

## Discussion

There is very limited information about the sex and age related differences in expression profile of human heart tissue in healthy people. We analyzed the gene expression profiles of 29 men and 17 women without a history of heart disease and identified 178 differentially expressed genes between the sexes. The majority of these genes demonstrated modest absolute fold-changes of 1.5–2.0 (77.5%) with greatest enrichment on autosomes 8 and 11 as well as the Y chromosome. A breakdown of these genes into biological processes revealed an over-representation of genes involved in inflammatory response in female heart tissue.

Our results are consistent with findings from a recent study on the expression of sexually dimorphic genes expressed in different human tissues, such as muscle, blood and brain, where more than 70% of sexually dimorphic genes displayed less than 1.2-fold change in expression. Greater fold changes (>2.0 fold) were seen in sex chromosome-linked genes. Our findings are also similar to those reported in a small microarray study (n = 6) of human explanted donor hearts (left ventricle) that identified 16 sexually dimorphic genes with more than 2-fold change with most differences linked to sex chromosomes [20].

Consistent with other reports in non-cardiac tissue [21], one of the important pathways differentially regulated in male and female heart in our cohort is the adaptive and innate immune system, which has been found to contribute to the development and progression of cardiovascular disease [22, 23] In our analysis, we found that male and female hearts differed significantly in the transcription of sex-linked and autosomal genes involved in the regulation of the immune system. Similar to findings from a previous small microarray study in healthy donor hearts (21), our cohort showed differential expression of two sex-linked genes DDX3Y and EIF1AY that encode for human male-specific minor histocompatibility antigens that contribute to inflammatory disease [16]. Of the autosomal genes related to inflammation, the greatest bias was found in females in the expression of TNFAIP3, a gene involved in immune and inflammatory responses signaled by such cytokines as TNF-alpha and IL-1 beta, or directly by pathogens via Toll-like receptors (TLRs) through terminating NF-kappaB activity [24]. The other important inflammatory genes with greater than 2-fold change in expression levels were CCL4, CX3CL1 and VCAM1[25–28]. Both CCL4 and CX3CL1 are chemokines that regulate the migration and adhesion of monocytes and lymphocyte and, thus, play an important role in left ventricular remodeling in response to injury [29]. VCAM-1, on the other hand, is expressed on the cytokine-activated endothelium, attracting neutrophils to damaged myocardium and mediating ischemia reperfusion injury [30]. Finally, we found that the most important transcription factors enriched in female heart were KLF4 (Kruppel-like factor 4) and NF-kappaB. While the role of KLF4, a regulator of pro-inflammatory signaling in the heart, has been well established in the development of vascular disease including atherosclerosis [31–33], a recent study has also shown that KLF4 is important in regulating cardiac hypertrophy and cardiac mitochondrial homeostasis [34, 35]. In addition to KLF4, we found that NF-kappaB was differentially expressed. NF-kappaB controls multiple processes, including immunity, inflammation, cell survival, differentiation and proliferation, and regulates cellular responses to stress, hypoxia, stretch and ischemia. NF-kappaB has been shown to influence numerous cardiovascular diseases including atherosclerosis, cardiac hypertrophy, and heart failure [36]. Although several studies have shown that estrogen and androgen receptors control different cardiovascular related pathways in a sex-specific manner, such as the synthesis of collagen and matrix-metalloproteinase [37–40], we did not find any differences in hormone receptor gene expression in heart tissue between male and females in our cohort. It appears that like other tissues, sex specific differences mediated by sex hormones is not related to differential gene expression of hormone receptors[41].

There are several limitations to this study. Because of the size of our cohort, we could not perform eQTL analysis. As additional samples become available, these analyses could be performed. We also did not include diseased hearts in our cohort. This is beyond the scope of this study and has been performed previously [42]. It would also be important to further validate our findings using quantitative real-time PCR as well as evaluating the sex differences on the proteomic level. Nevertheless, this study is the first to evaluate RNAseq data from normal male and female hearts and contributes to a better understanding of how differences in gene expression may affect cardiovascular health in men and women.

## Conclusion

In conclusion, gene expression in the human heart displayed evidence of sexual dimorphism similar to other somatic tissues. Notably, the female biased genes are highly enriched for genes involved in inflammatory response while no such pattern was seen for the male-biased genes, suggesting that the differences in cardiovascular disorder susceptibility between males and females are likely rooted from the sex-biased gene expression of the immune system.

## Supporting information

S1 Table Clinical data for samples from GTEX study. (XLSX)

S2 Table Differentially expressed genes between male and female heart samples (include all samples). (XLSX)

S3 Table Age biased genes in heart tissue. (XLSX)

S4 Table Transcription factor families with significant binding site enrichment in promoters of male- and female-biased genes. (XLSX)
